# Facilitating better postnatal care with women-held documents in The Gambia: a mixed-methods study

**DOI:** 10.1186/s12884-021-03902-6

**Published:** 2021-07-02

**Authors:** Tiffany Gooden, Lotta Gustafsson, Fides Lu, Faith Rickard, Alice Sitch, Carole Cummins, Kebba Manneh, Amie Wilson, Christine MacArthur, Semira Manaseki-Holland

**Affiliations:** 1 Institute of Applied Health Research, University of Birmingham, Edgbaston, Birmingham, UK; 2 University of Birmingham Medical School, Edgbaston, Birmingham, UK; 3 NIHR Birmingham Biomedical Research Centre, University Hospitals Birmingham NHS Foundation Trust and University of Birmingham, Birmingham, UK; 4 Kanifing General Hospital, Banjul, The Gambia

**Keywords:** Maternity care, Women-held documents, Discharge, Continuity of care, The Gambia, Low-income country, Mixed-methods, Handover

## Abstract

**Background:**

Women-held documents are a basic component of continuity of maternity care. The use and completion of women-held documents following discharge could improve treatment and care for postnatal women. Using a mixed-methods study design, we aimed to assess the number, type, quality and completeness of women-held discharge documents, identify factors contributing to document completeness and facilitators or barriers for effective use of the documents.

**Methods:**

Documents given to women at discharge from three hospitals in the Greater Banjul Area, The Gambia, were reviewed for content and quality. All women completed a questionnaire on the use of the documents. Poisson regression was used to estimate factors predicting document completion. Semi-structured interviews (*n* = 21) and focus groups (*n* = 2) were carried out with healthcare professionals (HCPs).

**Results:**

Nearly all (*n* = 211/212; 99%) women were given a document to take home. The most complete document (maternal record) had on average 17/26 (65%) items completed and 10% of women held an illegible document. None of the women’s sociodemographic or clinical characteristics predicted document completeness. The following facilitators for effective use of documents were identified from the women’s responses to the questionnaire and interviews with HCPs: 94% of women thought written information is important, 99% plan to have postnatal check-ups and 67% plan to use their documents, HCPs understand the importance of the documents and were familiar with the document’s use and content. The following barriers for effective use of documents were identified: HCPs had too many women-held documents to complete at discharge, there is no national protocol and HCPs think women do not understand the documents due to a lack of education and that women often lose or forget their documents.

**Conclusions:**

Women-held documents are well established in The Gambia; though quality and completeness needs improving. Future research should determine the impact of using only one document at discharge, protocols and training on completeness, among other outcomes, and on ways to ensure all women are using the documents for their postnatal care.

**Supplementary Information:**

The online version contains supplementary material available at 10.1186/s12884-021-03902-6.

## Background

Approximately 99% of global maternal deaths occur in low- and middle-income countries (LMICs), with 66% occurring in sub-Saharan Africa [1]. The Gambia is estimated as having one of the highest maternal mortality rates worldwide of 597 per 100,000 live births [1]. Reducing maternal mortality is in line with Sustainable Development Goals but can be challenging in resource-limited settings [2]. Insufficient access to healthcare is a major contributor to poor outcomes in rural areas [3]. However, as a result of great efforts for improving access and utilisation of healthcare, poor quality care has superseded inequity as the leading cause of mortality in LMICs [3].

Continuity of care is a key element in providing high quality, safe and coordinated care; this includes handover of patient information between healthcare professionals (HCPs) and their patients [4]. Poor continuity of care is partially to blame for maternal deaths in LMICs, where it is not uncommon for women to visit multiple healthcare facilities for their postnatal care [5–7]. One way to improve continuity of maternal care is by capturing detailed information on women-held documents. These documents can provide HCPs at different facilities with the necessary information to provide appropriate and timely management of postnatal care [8]. The World Health Organisation (WHO) considers the use of women-held documents a basic component of continuity of maternal care [8]. HCPs in LMICs have reported improved health outcomes resulting from women-held documents [7]. Additionally, such documents increase the likelihood of women initiating postnatal care through check-up appointments [9]. For women-held documents to be effective sources of information, HCPs must complete them and the women must take them to their appointments.

Whilst women-held documents are used in most countries [8], a recent systematic review found no studies that report completion of such documents or predictors of complete written information at the time of discharge [10]. Women are particularly at high risk following birth [8]. In LMICs, women-held documents often serve as the only medical records available [11]. Therefore, assessing such documents and identifying contributing factors to their completion and use are vital steps toward improving handover of essential information after birth for improving management of postnatal complications [7, 9].

The primary aim of this study was to assess the number, type, completeness and quality of women-held documents on discharge from maternity units in The Gambia, identify any factors associated with completeness and explore context-specific barriers and facilitators for the document’s effective use.

## Methods

This study was a component of a larger study investigating the use of women-held documents upon arrival for maternity care in The Gambia [12]. The reporting of our research was guided by the Minimum Standards of Reporting Checklist and the cross-sectional Strengthening the Reporting of Observational Studies in Epidemiology (STROBE) guidelines. The Consolidated Criteria for Reporting Qualitative Research (COREQ) checklist can be found in Additional file 1.

### Setting and Design

Three maternity hospitals in the Greater Banjul Area were the setting of this cross-sectional mixed-methods study [13]. Each hospital’s characteristics are described in Additional file 2. Women were surveyed, documents were examined and HCPs interviewed between January and March 2018. Written informed consent was obtained by all women in the study with signature or thumbprint if the woman was illiterate.

### Quantitative component

Sample size calculations for the quantitative element were carried out for a linked study [12]. Calculations were based on an unknown population size and an assumption that 80% of women would have documents [14]. For the linked study, a minimum sample size of 243 women was needed to achieve a sufficiently precise estimate (± 5% using a 95% confidence level) [12].

Researchers (LG, FL and FR) and Gambian translators were trained in the use of the questionnaire and assessment of documents. They rotated around the hospitals across an even distribution of weekdays and weekends to generate a representative sample and reduce observer bias. They approached all women being discharged on each day they were present (normally between 9am and 1 pm).

The inclusion criteria comprised women aged 16 and older being discharged from the hospitals who had a live birth during their stay. Women were excluded if they had partaken in any Medical Research Council (MRC) study or were unable to speak English or one of the three major tribal languages of Mandinka, Wolof or Fula. Those recruited gave informed consent with thumbprint or signature.

All women included had their documents assessed for document type, completeness and quality. Content quality was assessed by determining legibility; a document was considered illegible if both the researcher and translator were unable to read the content. Document completeness was assessed using a 26-item checklist developed using a combination of the government-issued maternal record used in The Gambia and the 2015 WHO guide for essential practice in pregnancy, childbirth, postpartum and newborn care [15]. Final checklist items were agreed by senior co-authors.

To identify any facilitators and barriers for effectively using women-held documents from the women’s perspective, data on the women’s opinions and plans for using their women-held documents after discharge were collected with a questionnaire of close-ended questions (Additional file 3). The questionnaire was adapted from handover and discharge studies in Mongolia [16] and India [17] and were verbally administered in the women’s local languages.

Data was analysed using SPSS version 25.0 (IBM, Armonk, NY, USA). Descriptive statistics were used to describe the women’s characteristics, number, content and quality of documents and questionnaire responses. Poisson regression was used to assess for any factors associated with the number of completed items (maximum 26 per woman), adjusting for potential confounding factors. A *P*-value of less than 0.05 was considered statistically significant. The dependent variable was the number of items completed for each woman after combining all documents she held. Covariates entered into the model were determined based on clinical importance and evidence on which variables are associated with quality of written documentation [16, 17]. Occupation was agreed to be the best representation of socioeconomic status and was therefore used in the model along with age, education, travel time to hospital, complicated/normal birth (defined in Additional file 4), number of antenatal visits and literacy. The location of birth (hospital 1, 2 and 3) was added to the regression model as a fixed effect.

### Qualitative component

To identify any facilitators and barriers for effectively using women-held documents from the HCPs perspective, focus group discussions (FGDs) and one-on-one semi-structured interviews (SSIs) were undertaken. The qualitative component was conducted in parallel with the quantitative component at the same hospitals. A purposive sampling approach was used. Nurses, midwives and doctors on the maternity wards were invited to participate in either FGDs or SSIs based on their availability; none participated in both. Recruitment continued until thematic saturation was achieved [18].

A researcher (FL), from Chinese background and raised in the UK with no prior exposure to African setting, and trained in qualitative research conducted the interviews in English (all HCPs were fluent) at the hospital sites. At the time of the study, the interviewer was a medical student studying an intercalated BMedSci degree in Public Health and Population Sciences in a UK university. No prior relationship was established between the interviewer and the HCPs being interviewed. All interviews were conducted in person, recorded and anonymously transcribed verbatim. All FGDs and most SSIs were conducted in a quiet, private room; however, a few SSIs took place at the nurses’ station when the nurse being interviewed was the only staff member on the ward. The interview topic guide was piloted prior to the FGDs and SSIs (Additional file 5). Fifteen to thirty minutes were needed for the SSIs whereas the FGDs took twenty to forty minutes. Field notes were taken immediately after each FGD and SSI.

Inductive thematic analysis based on Braun and Clarke’s six-step approach [19] was undertaken for analysis. A different researcher (TG), from the US working as a researcher in the UK with a masters in international public health and no prior exposure to the Gambian setting, performed line-by-line coding on all transcripts. Data was coded for facilitators and barriers for effective use of women-held documents at discharge. Subsequently, themes and sub-themes were identified, refined and approved by senior authors in an iterative process. Convergent triangulation was used to combine and discuss quantitative and qualitative results [20].

## Results

### Quantitative results

**Fig. 1:**
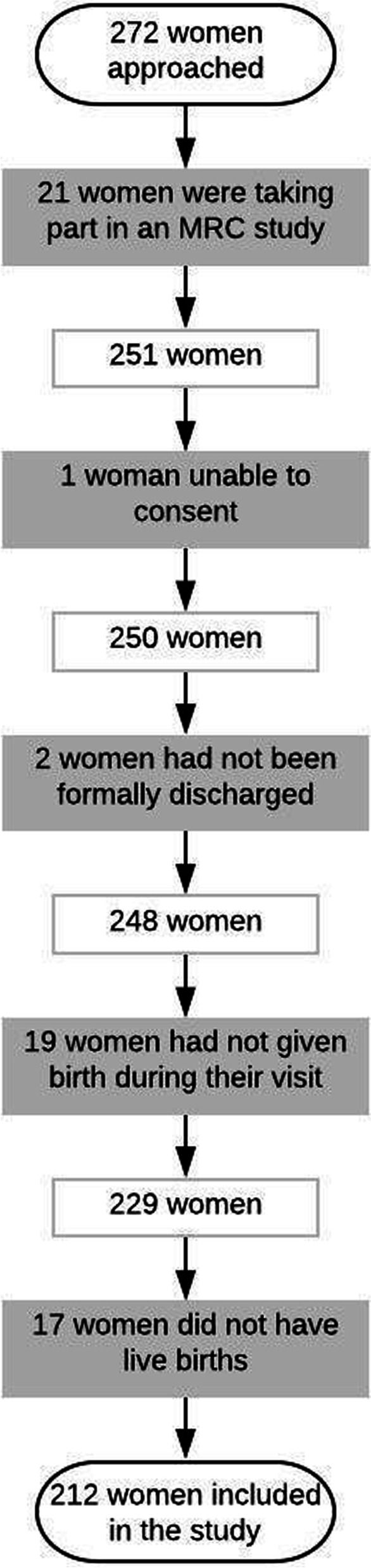
Sample recruitment flowchart

All 272 women approached agreed to partake. However, twenty-one were involved in an MRC study, one lacked capacity to consent, two had not been formally discharged, nineteen had not given birth and seventeen did not have a live birth. These sixty women were subsequently excluded, leaving 212 women eligible for the study (Fig. 1). Additional file 6 shows women’s demographic and pregnancy characteristics. Across the three hospitals, women differed in the travel time to the hospital, the type of transportation they took and whether or not they had a complicated birth.

#### Number and type of documents

**Fig. 2:**
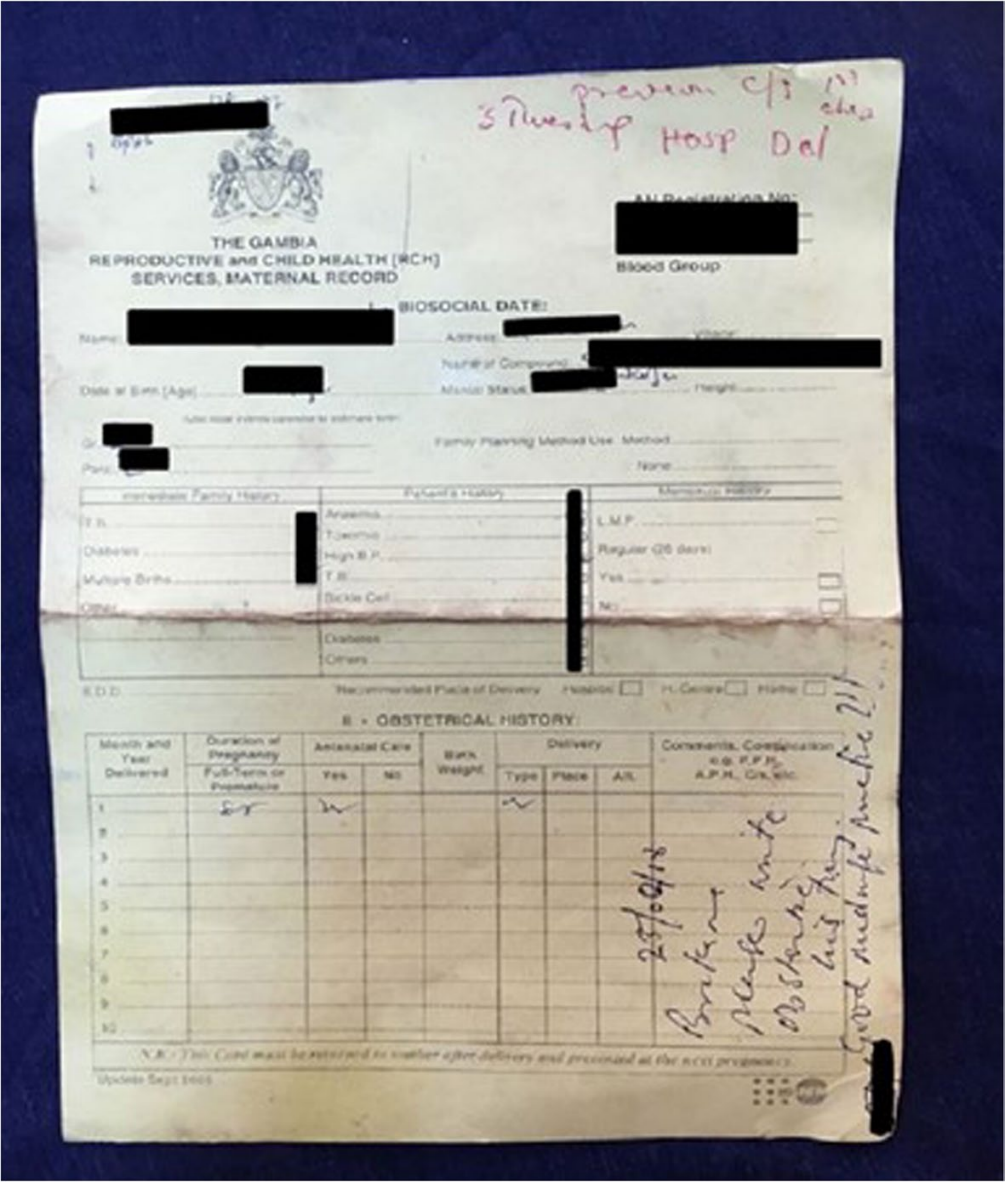
Front side of the government-issued maternal record

**Fig. 3:**
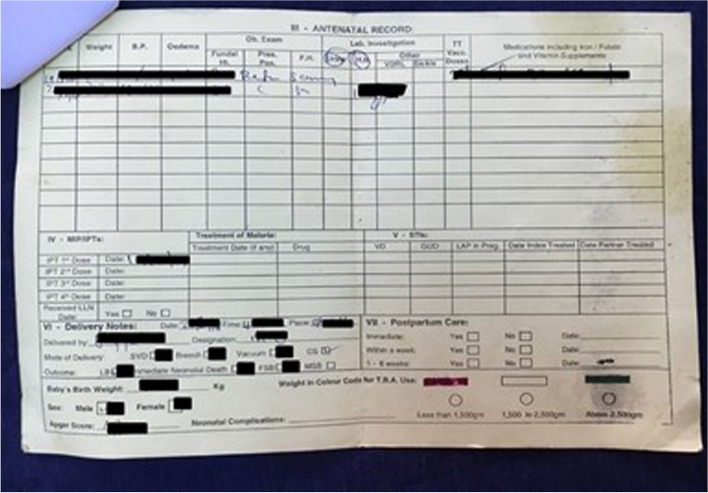
Back side of the government-issued maternal record

All documents were assessed by researchers. One woman (0.5%) did not have a document to assess. Seventy-four percent (*n* = 157) held more than one document. The government-issued maternal record (Figs. 2, 3) was most commonly held (*n* = 207; 98%). Prescription cards were the second most common document held (*n* = 111; 52%). Eighty women (38%) had a discharge card or checklist; however, the checklist was used at only one hospital (44 women from this hospital had the checklist; 54%). Thirty-one women (15%) were provided with pieces of paper, referral forms and miscellaneous documents such as ultrasound reports.

#### Quality and completion of documents and associated factors for completeness

Twenty-two women (10%) had at least one illegible document and two (1%) held no legible document. The maternal record had 17 (65%) items completed on average, the highest of any document (Additional file 7). Discharge checklists had the second highest average of items complete, with 9 (35%) items complete on average. All but five items were better recorded on the maternal records compared to any other document: date of discharge, name of staff that issued the document, date of next appointment, and mother’s vaccinations and medications.

Of the women that had a document at discharge (*n* = 211; 99%), the number of items completed ranged from 5 to 22 (mean = 17; standard deviation (SE) = 3.04). Regression results indicate that no sociodemographic or clinical characteristic tested were associated with the proportion of items complete (Table 1). However, the following variables had an Incident Risk Ratio (IRR) greater than 1: age (age 30 + IRR 1.039, 95% CI 0.962–1.123; age 21–29 IRR 1.031, 95% CI 0.958–1.110), time to hospital (under 1 h IRR 1.028, 95% CI 0.961–1.099), literacy (literate IRR 1.058, 95% CI 0.997–1.123) and complicated birth (complication IRR 1.055, 95% CI 0.980–1.136). The following variables had an IRR less than 1: education (none or did not complete primary school IRR 0.974, 95% CI 0.898–1.055), occupation (retail job IRR 0.993, 95% CI 0.934–1.055) and number of antenatal visits (more than 3 IRR 0.945, 95% CI 0.890–1.003). All assumptions of a Poisson regression were met for the analysis.

**Table 1:** Regression results for predictors of completion

**Respondent Variable**	***P*-value**	**IRR (95% CI)**
Age		
30 +	.330	1.039 (.962, 1.123)
21–29	.417	1.031 (.958, 1.110)
Under 20		1^a^
Education		
None or did not complete primary school	.515	.974 (.898, 1.055)
Completed junior secondary school	.608	.981 (.911, 1.056)
Completed senior secondary school	.819	.992 (.922, 1.066)
Islamic or other		1^a^
Occupation		
Retail	.812	.993 (.934, 1.055)
Other	.374	.965 (.893, 1.043)
Housewife		1^a^
Time to hospital		
Under 1 h	.425	1.028 (.961, 1.099)
More than 1 h		1^a^
Number of antenatal visits		
More than 3 visits	.064	.945 (.890, 1.003)
0 to 3		1^a^
English literacy		
Literate	.064	1.058 (.997, 1.123)
Illiterate		1^a^
Complicated birth		
Yes	.151	1.055 (.980, 1.136)
No		1^a^
Hospital		
1	.773	.988 (.913, 1.070)
2	.743	.990 (.933, 1.051)
3		1^a^

^a^ An IRR of 1 indicates the reference category of the predictor variable

#### Women’s opinions and plans for using the women-held documents

Additional file 8 shows questionnaire responses, analysed by whether the woman had a complicated birth. Most women (*n* = 211; 99%) planned to see an HCP for a postnatal check-up. Over half (*n* = 125; 59%) planned to visit the same hospital for their check-up (45 women (46%) with a complicated birth and 80 women (71%) with a normal birth). Forty women (19%) stated they would not be able to tell the HCP at their check-up what happened during birth, whereas sixty-seven percent (*n* = 142) said they would use documents HCPs had given them (80 women (80%) with a complicated birth and 62 women (55%) with a normal birth). Most women thought a written explanation of what happened during their visit was important (*n* = 199; 94%). The most common reason given (*n* = 175; 83%), was that it helped them when attending postnatal services whereas the next highest reason (*n* = 24; 11%) was that it made them feel confident about what to do next.

### Qualitative results

Twenty-one HCPs (8 doctors, 8 midwives and 5 nurses) participated in SSIs whilst nine participated in two FGDs. All HCPs approached agreed to take part; however, some were unable to due to workload. The demographic details of the participants interviewed are included in the Additional file 9 and published elsewhere [12]. Two themes identified were related to the effective use of women-held documents for continuity of post-delivery care: HCPs completing and handing over documents to women, and women’s ability to understand the documents. Divided into facilitators and barriers, themes and sub-themes are presented in Additional file 10 with supporting quotations.

#### HCPs ability to effectively use women-held documents

The first theme considers the role of HCPs in completing the documents and effectively handing them over to women. Identified facilitators included the HCPs knowledge and consistent use of women-held documents, including the government-issued maternal record (referred to as antenatal card in the interviews). HCPs understood the importance of the women-held documents, knew they were to be used for the postpartum period and knew from memory the basic items to record; however, two HCPs believed they were only used for normal births. One doctor explained:

“…the documents need to be kept in their homes. In their subsequent pregnancies, they go with [these documents] so that when they are starting their antenatal, that doctor or nurse will have an idea of what has happened in the past pregnancies and what was done and how does it end up because sometimes it is difficult to get those information.”

Barriers identified included HCPs having too many discharge documents to complete and lacking the time to complete them due to understaffed hospitals. The final barrier identified was the lack of protocols on written documents; it was mentioned there is currently no national guidelines on which documents to complete and give to women at discharge. HCPs felt this contributed to inconsistencies in the documents used. A midwife stated:

“…a standardised protocol that we can go through and verbally present it to the others, they might appreciate it, we can adapt it and work towards it.”

#### Women’s ability to effectively use women-held documents

The second theme—the women’s ability to utilise the documents—identified two barriers but no facilitators. From the HCPs’ perspectives, one barrier was the women’s education level. Some HCPs believed illiteracy to be the problem and others mentioned that uneducated women did not understand the information given to them. One midwife stated:

*“*…there is a lot of women who didn’t go to school and are illiterate, no matter how much you talk to them, they still when they go home, their mothers, their aunties, they still influence them with their cultures and their beliefs.”

Several HCPs also stated that women often lose or forget their documents. HCPs provided the following rationales for this: women think the documents are unimportant, they have too many loose documents and they forget them when they are rushing to the hospital. A doctor explained:

“They leave all their medica-the other cards at home, and then you have to try to trace the folder back to-to know what exactly was done … sometimes, they have just an urgent thing or an urgent- er a new emerging problem and if they rush to the hospital, they forget everything. I think an emphasis should be made completely that any time that you are visiting, even if it is outside the normal appointment, you should bring along all the documents. Er it will help to improve, improve the service.”

## Discussion

Women-held documents are the commonest means for information transfer between antenatal, birth and postnatal visits for women in LMICs. They facilitate continuity of care between a range of HCPs and ultimately better outcomes for women and newborns. We found that 99% of women held one or more document following birth at three hospitals in The Gambia’s capital city. The government-issued maternal record was most commonly used, followed by prescription cards, discharge cards, discharge checklists and other miscellaneous documents. However, information transfer was hampered in instances where for example ten percent of women had illegible documents and completeness of information was sub-optimal. This inadequate provision of documented information was not associated with the sociodemographic or clinical profile of women. Several facilitators for effective use of women-held documents were identified that could form the basis of improving documentation: 99% of women planned to have postnatal check-ups and most said they would use their documents to explain what happened during birth; 94% of women thought having written information was important; HCPs were familiar with such documents, understood their importance, knew the information that was to be recorded on them and knew they covered the postnatal period. Barriers needing to be addressed for effective use of women-held documents included HCPs having too many documents to complete, having no national protocol and HCP’s perspectives which may sub-consciously impact the completeness of documents (HCPs said they thought women did not understand the documents due to a lack of education and they often lose or forget their documents).

Most women had more than one document (*n* = 157; 74%), supporting the comments made from HCPs regarding the various documents they must complete and give to women. Having only one document to complete and explain to women could save HCPs valuable time, thus increase time for patient care, the likelihood of the correct document being taken to postnatal appointments by women and potentially improve completeness of information. We found that the maternal record was the most commonly used and most complete women-held document. Two HCPs thought the maternal record was only used for normal births, though the quantitative data disputes this. Four of the five items that were recorded less on the maternal record were better recorded on the discharge checklist; however, the checklist was not universally given to women. Based on these findings, it would be practical to consolidate all necessary items onto the maternal record for it to be the only women-held document provided after discharge. However, whether this would improve completeness of documents and clinical outcomes would need to be investigated in future research.

Our results complement the existing literature on women’s opinions and use of women-held documents [9, 10, 12, 21]. Most women in our study said a written explanation on what happened during birth was important and they would use the document(s) given to them. However, 19% of women in our study said they did not know how they would inform the next HCP about the birth (i.e. they did not mention they would use the documents). Although there is an on-going need to improve women’s understanding and use of maternity cards, it is concerning that HCPs think women do not understand the documents and find these documents unimportant. It is possible that incomplete documentation is impacted by this perception, compounded by HCPs’ views on staff limitations and a lack of protocols. Completeness of documents is a low cost and potentially high impact intervention to improve women’s health. If key information is missing from documents, important clinical needs of the woman may be overlooked or inappropriate care may be given which may be dangerous for the woman and child. Handover of such information is a vital element of patient safety, as driven by WHO in recent years [22].

In addition to patient safety, women-held documents are fundamental for quality, patient-centred maternal care [8]. They can improve communication between women and HCPs, improve continuity of care between a range of HCPs and empower women, making them feel more confident and in control of their care [9, 23–26]. Interventions need to be designed and informed by contextual formative research for improving women’s use of documents following discharge. To improve HCPs’ ability to provide women with consistently completed documents, there is a need for protocols and proformas with minimum data clearly outlined, training on completeness and importance of documentation, supervision and motivational elements of such health system interventions that enable behaviour change and change in organisational culture. Such interventions would allow for clarifications to be made to HCPs that women rarely forget to bring their documents to healthcare appointments in The Gambia and elsewhere [10, 12, 27], they generally find them important and that women-held documents have a wider impact than just continuity of care.

A major strength of this study is the mixed-methods design. We were able to explore women’s and HCP’s perspectives on women-held documents (via a questionnaire and interviews, respectively); thus, enabling facilitators and barriers to be identified in the effective use of women-held documents. The qualitative component was particularly useful for adding context to the quantitative findings. However, this study had some limitations to mention. Due to resources and time constraints, it was not possible to collect qualitative data from the women. Although we gained insight from the quantitative questionnaire, the women’s in-depth view of women-held documents is missing. The criteria used to assess document completeness was partially based on the maternal record. Consequently, this may have positively influenced the completeness of maternal records; however, the criteria were also guided by WHO recommendations. Results may be less generalisable to rural Gambia, although all rural areas around Banjul referred women to these hospitals. The time it took for women to reach the hospital did not predict completeness and 99% of woman held a document, suggesting that use and completeness would not differ between urban inhabitants near the hospitals and rural inhabitants further away. The minimum sample requirement of 243 was calculated for a linked study [12] and may indicate that the quantitative element of this study was underpowered. However, the original calculation was based on 80% of women having documents [12] and our study found that 99% of women had documents; thus, it is likely our study was sufficiently powered for our specified aims.

## Conclusions

Most women in The Gambia are given at least one document to take home at discharge. HCPs understand the importance of women-held documents and their intended use; however, completeness and quality of the documents require improvements. None of the women’s sociodemographic or clinical factors predicted document completeness. However, we identified various barriers for effectively using the documents that might be contributing factors: HCPs have limited time to complete the multiple women-held documents required at discharge, a protocol is lacking on what document(s) to complete and what information to include and HCPs think that women do not understand the documents and that they often lose them. It is recommended that further research investigates the effectiveness of using only one women-held document at discharge and a national protocol be subsequently developed. The protocol should be complemented with training where HCPs are informed that women rarely forget their documents, they think written information is important and most women intend to use the documents during their postnatal care. Future research should focus on effective ways to enhance women’s use of the documents after discharge; thus, to ensure all women receive the benefits of having women-held documents. Utilising the aforementioned facilitators and barriers to improve use of women-held documents at discharge could facilitate safer transition of women between healthcare facilities and increase effectiveness of the management of postpartum complications, thereby contribute to the international aim in reducing global maternal mortality.

## Supplementary Information

**Additional file 1.** ISSM_COREQ_Checklist. Completed COREQ checklist for the qualitative component of the study.

**Additional file 2.** Hospital background information. Additional information on the hospitals used for the setting of this study.

**Additional file 3.** Questionnaire used to collect data on women’s characteristics, the documents they held and their opinion of the documents.

**Additional file 4.** Definition of complicated birth. The criteria used to define a complicated birth in this study.

**Additional file 5.** Interview topic guide for FGDs and SSIs. Topic guide for focus discussion groups and semi-structured interviews used for collecting the qualitative data in this study.

**Additional file 6.** Women’s background and pregnancy characteristics; figures presented as N (%). Baseline figures for the women included in this study including demographics.

**Additional file 7.** Content quality and completeness of documents; figures presented as N (%). Results from the assessment of women-held documents.

**Additional file 8.** Questionnaire responses. Results from the questionnaire each woman completed.

**Additional file 9.** Qualitative participant demographics. Demographic data of the healthcare professionals that participated in the focus group discussions and semi-structured interviews.

**Additional file 10.** Barriers and facilitators for effective handover of information from HCPs to other HCPs and women. Results from the focus groups discussions and semi-structured interviews.

## Data Availability

The dataset supporting the conclusions of this article is available in the figshare repository [13199942; https://figshare.com/articles/dataset/Full_dataset_Maternity_cards_in_The_Gambia_xlsx/13199942].

## Abbreviations

LMICs: Low- and middle-income countries
HCPs: Healthcare professionals
WHO: World Health Organisation
MRC: Medical Research Council
FGDs: Focus group discussions
SSIs: Semi-structured interviews
IRR: Incident risk ratio
