# Supplementary material for: Facilitating better postnatal care with women-held documents in The Gambia: a mixed-methods study
Source: BMC Pregnancy Childbirth. 2021 Jul 2;21:479. doi: 10.1186/s12884-021-03902-6 (PMC8254330; doi:10.1186/s12884-021-03902-6)
Supplement: Supplementary file 2 — Additional file 2. Hospital background information. Additional information on the hospitals used for the setting of this study. [file 12884_2021_3902_MOESM2_ESM.docx]

Additional file 2: Hospital background information(12)

Antenatal care is provided as part of the Maternal, Child health and Family Planning program (MCHFP) and can take place at a variety of health facilities ranging from mobile health posts and local health centres to the tertiary hospital in Banjul. Some health centres have birth facilities, others only provide antenatal care and tell mothers to go to hospital to deliver.

Primary healthcare centres can refer women to any of the three hospitals, normally the closest maternity unit geographically. Women experiencing complications in hospitals in provinces further inland (‘upcountry’) are sometimes referred to Hospitals 2 or 3.

| **Hospital 1** | **Hospital 2** | **Hospital 3** |
| --- | --- | --- |
| A maternity specific facility in a more community setting offering emergency obstetric care | A growing hospital in the centre of the urban coastal area offering emergency obstetric care | The only official tertiary hospital in The Gambia, providing specialist care and the largest numbers of doctors of any of the hospital in The Gambia. Offers specialist emergency obstetric care. |
| Low referral rate | It is a referral centre for many of the surrounding health centres | Highest referral rate |
| Refers complex cases to Hospital 2 and 3 | Still refers the most complex cases to hospital 3 | Receives the most complex cases |
| 33 beds (10 on high dependency unit) | 28 beds (no high dependency unit) | 56 beds (8 on high dependency unit) |
| Doctors 4  Midwives 12  Nurses 20 | Doctors 5  Midwives 19  Nurses 12  Nursing Attendants 12 | Doctors 33  Midwives 27  Nurses 29 |
