# Supplementary material for: Facilitating better postnatal care with women-held documents in The Gambia: a mixed-methods study
Source: BMC Pregnancy Childbirth. 2021 Jul 2;21:479. doi: 10.1186/s12884-021-03902-6 (PMC8254330; doi:10.1186/s12884-021-03902-6)
Supplement: Supplementary file 4 — Additional file 4. Definition of complicated birth. The criteria used to define a complicated birth in this study. [file 12884_2021_3902_MOESM4_ESM.docx]

Additional file 4: Definition of complicated birth

Complicated birth includes (not an exhaustive list):

- any birth not defined as spontaneous vaginal delivery
- pre-eclampsia
- obstruction
- placenta praevia
- placental abruption
- post-partum haemorrhage
- still-birth
- Intra-uterine fetal death
- Fetal distress
- Uterine rupture
- Premature rupture of membranes (PROM)
- Severe anaemia
