# Supplementary material for: Facilitating better postnatal care with women-held documents in The Gambia: a mixed-methods study
Source: BMC Pregnancy Childbirth. 2021 Jul 2;21:479. doi: 10.1186/s12884-021-03902-6 (PMC8254330; doi:10.1186/s12884-021-03902-6)
Supplement: Supplementary file 5 — Additional file 5. Interview topic guide for FGDs and SSIs. Topic guide for focus discussion groups and semi-structured interviews used for collecting the qualitative data in this study. [file 12884_2021_3902_MOESM5_ESM.docx]

Additional file 5: Interview topic guide for FDGs and SSIs(12)

# Interview Topic Guide for FGDs and SSIs

**Introduction:**

- State participant ID at the start
- Confirm verbal consent from participant/s after written consent is obtained
- Welcome participant/s and thank them for taking part

**[Start Interview]**

- Tell me what you understand by clinical handover.
- (Researcher to define this after exploration of what the interviewees understand – it is the transfer of patient information and patient responsibility to the next group of care providers and is critical for ensuring continuity of patient care and safety)
- When do you think these are most important?

**I would like to discuss different types of clinical handover – first let us start with shift-to-shift changes that take place on the ward:**

- Tell me what happens when passing on women’s clinical information to the next group of staff who start work as you finish your shift on wards.
  - Tell me more about what information you pass on.
- When you start your shift, do you feel like you receive adequate information from the last shift to be able to facilitate proper care for the women on the ward?
  - What do you do if you feel you don’t know enough about a woman’s condition you have to care for? (This could be at the time of shift handover, when the work progresses or when a patient has a crisis or needs special care)
- Is there something different that is done when handing over the care of a woman that is considered to be a ‘high-risk’ patient?
- Who is normally involved in handover?
- In your opinion, what barriers are there that prevent good / effective handover at shift times?
- Are there any problems with the handing over at the moment?
- Would you like to see any changes? If so, what would they be?
- Did you have any training about this?
- Do you know of any protocols, guidelines or systems that could be followed for handover of clinical information at shift times? If yes, how are these implemented?
- How do you think handover of care can be improved at shift times that is feasible here in The Gambia?
- (Researcher to note possible methods: quiet and private space/room, an admission book, a board on the ward, an SBAR or SHARING type form per patient etc.)
- Do you think the implementation of a certain layout for handover that every clinician follows would be beneficial?

**Now I would like to discuss the time when the women first arrive on the ward, either from referral from another team of staff from primary care or if they walk in themselves (self-referred):**

- In your experience/perception, do most of the women come for a ‘normal’ delivery when they are having contractions and in labour or are they high-risk patients referred to you or have had a problem and walk in themselves because they are worried?
- Please tell me what kind of documentation the women bring when they first come – any document that tells you about their pregnancy, previous pregnancy or why they have come to the maternity ward?
- Specifically, if they come after referral, who usually refers them?
- (If the answer is the hospital outpatients, then ask who refers them to the outpatient department)
- For women with normal labour (with a maternity card) and those with danger signs or risk factors, do you feel like you receive adequate information from primary care / the community healthcare professionals to be able to facilitate proper care for the women on the ward?
- What do you think constitutes good practices for handover of clinical information for referral of a maternity patient to the hospital from the community?
- In your opinion, what barriers are there that prevent good / effective handover for referral of a maternity patient to the hospital from the community?
- Did you have any training about this?
- Do you know of any protocols, guidelines or systems that could be followed for handover of clinical information for referral of a maternity patient to the hospital?
- How do you think handover of care can be improved for referral of a maternity patient to the hospital that is feasible here in The Gambia?
- Do you think the implementation of a certain layout for handover that every clinician follows would be beneficial?

**Now about the discharge time:**

- What papers/documents/explanation do you give the women about their admission or summary of their labour when they are discharged from the ward?
  - (Researcher to explore verbal and written instructions including notes in the maternity card held by the mother)
- Is there a communication system with the community/primary care midwives or doctors about the delivery or admission – if yes what do you do?
- What do you think constitutes good practices for handover of clinical information to the women or for the next healthcare provider for discharging the women and babies from the hospital?
- In your opinion, what barriers are there that prevent good / effective handover of clinical information to the women or for the next healthcare provider for discharging the women and babies from the hospital?
- Did you have any training about this?
- Do you know of any protocols, guidelines or systems that could be followed for handover of clinical information to the women or for the next healthcare provider for discharging the women and babies from the hospital?
- How do you think discharge handover of clinical information can be improved for the patient or for the next healthcare provider for discharging the women and babies from the hospital?
- Do you think the implementation of a certain layout for discharging information for handover that every clinician follows would be beneficial?

**Conclusion:**

- Ask participant/s if there are any other issues they would like to raise
- Thank participant/s

**[End of interview]** Make notes and memos after FGD or SSI is conducted.
