# Supplementary material for: Facilitating better postnatal care with women-held documents in The Gambia: a mixed-methods study
Source: BMC Pregnancy Childbirth. 2021 Jul 2;21:479. doi: 10.1186/s12884-021-03902-6 (PMC8254330; doi:10.1186/s12884-021-03902-6)
Supplement: Supplementary file 6 — Additional file 6. Women’s background and pregnancy characteristics; figures presented as N (%). Baseline figures for the women included in this study including demographics. [file 12884_2021_3902_MOESM6_ESM.docx]

| **Additional file 6.** Women’s background and pregnancy characteristics; figures presented as N (%) | | | | |
| --- | --- | --- | --- | --- |
| **Background Characteristics** | **Hospital 1 [n=54]** | **Hospital 2 [n=76]** | **Hospital 3 [n=82]** | **Total [n=212]** |
| ***Age*** |  |  |  |  |
| Under 20 | 9 (16.7) | 10 (13.2) | 15 (18.3) | 34 (16.0) |
| 21-29 | 23 (42.6) | 33 (43.4) | 42 (51.2) | 98 (46.2) |
| 30+ | 22 (40.7) | 33 (43.4) | 25 (30.5) | 80 (37.7) |
| ***Education*** |  |  |  |  |
| None / incomplete primary | 11 (20.4) | 19 (25.0) | 10 (12.2) | 40 (18.9) |
| Islamic or other | 16 (29.6) | 24 (31.6) | 23 (28.0) | 63 (29.7) |
| Completed primary | 4 (7.4) | 2 (2.6) | 1 (1.2) | 7 (3.3) |
| Junior Secondary | 11 (20.4) | 14 (18.4) | 17 (20.7) | 42 (19.8) |
| Senior Secondary or higher | 12 (22.2) | 17 (22.4) | 31 (37.8) | 60 (28.3) |
| ***Tribe*** |  |  |  |  |
| Mandinka | 18 (33.3) | 29 (38.2) | 30 (36.6) | 77 (36.3) |
| Fula | 11 (20.4) | 15 (19.7) | 19 (23.2) | 45 (21.2) |
| Wolof | 11 (20.4) | 15 (19.7) | 19 (23.2) | 45 (21.2) |
| Other | 14 (25.9) | 17 (22.4) | 14 (17.1) | 45 (21.2) |
| ***Occupation*** |  |  |  |  |
| Housewife | 35 (64.8) | 43 (56.6) | 59 (72.0) | 137 (64.6) |
| Retail | 9 (16.7) | 22 (28.9) | 11 (13.4) | 42 (19.8) |
| Other | 10 (18.5) | 11 (14.5) | 12 (14.6) | 33 (15.6) |
| ***Time to hospital*** |  |  |  |  |
| Under 1 hour | 34 (63.0) | 50 (65.8) | 81 (98.8) | 165 (77.8) |
| 1 hour and over | 20 (37.0) | 26 (34.2) | 1 (1.2) | 47 (22.2) |
| ***Transport*** |  |  |  |  |
| Walked | 3 (5.6) | 1 (1.3) | 7 (8.5) | 11 (5.2) |
| Taxi/Gelli | 25 (46.3) | 63 (82.9) | 75 (91.5) | 163 (76.9) |
| Ambulance | 26 (48.1) | 12 (15.8) | 0 (0.0) | 38 (17.9) |
| ***Adults in house (apart from self)*** |  |  |  |  |
| 0-2 | 20 (37.0) | 32 (42.1) | 25 (30.5) | 77 (36.3) |
| 3-6 | 21 (38.9) | 27 (35.5) | 29 (35.4) | 77 (36.3) |
| 7+ | 13 (24.1) | 17 (22.4) | 28 (34.1) | 58 (27.4) |
| ***Children in house*** |  |  |  |  |
| 0-2 | 16 (29.6) | 22 (28.9) | 18 (22.0) | 56 (26.4) |
| 3-6 | 12 (22.2) | 22 (28.9) | 32 (39.0) | 66 (31.1) |
| 7+ | 26 (48.1) | 32 (42.1) | 32 (39.0) | 90 (42.5) |
| ***Rooms*** |  |  |  |  |
| 1-2 | 17 (31.5) | 29 (38.2) | 30 (36.6) | 76 (35.8) |
| 3-4 | 12 (22.2) | 17 (22.4) | 11 (13.4) | 40 (18.9) |
| 5+ | 25 (46.3) | 30 (39.5) | 41 (50.0) | 96 (45.3) |
| ***Live children*** |  |  |  |  |
| 0-2 | 29 (53.7) | 42 (55.3) | 45 (54.9) | 116 (54.7) |
| 3-4 | 14 (25.9) | 15 (19.7) | 21 (25.6) | 50 (23.6) |
| 5+ | 11 (20.4) | 19 (25.0) | 16 (19.5) | 46 (21.7) |
| ***Address*** |  |  |  |  |
| Combo/Banjul/Kanifing | 28 (51.9) | 29 (38.2) | 77 (93.9) | 134 (63.2) |
| West Coast | 14 (25.9) | 46 (60.5) | 5 (6.1) | 65 (30.7) |
| Provinces (LRR, North Bank, CRR, URR) | 12 (22.2) | 1 (1.3) | 0 (0.0) | 13 (6.1) |
| ***Structure of house*** |  |  |  |  |
| Brick and tiles | 5 (9.3) | 8 (10.5) | 3 (3.7) | 16 (7.5) |
| Mud/sand and corrugate | 9 (16.7) | 12 (15.8) | 3 (3.7) | 24 (11.3) |
| Cement and corrugate | 39 (72.2) | 56 (73.7) | 76 (92.7) | 171 (80.7) |
| Other | 1 (1.9) | 0 (0.0) | 0 (0.0) | 1 (0.5) |
| ***English literacy*** |  |  |  |  |
| Literate | 30 (55.6) | 43 (56.6) | 55 (67.1) | 128 (60.4) |
| Illiterate | 24 (44.4) | 33 (43.4) | 27 (32.9) | 84 (39.6) |
| **Pregnancy Characteristics** |  |  |  |  |
| ***Complicated birth^a^*** | | | | |
| *Yes* | 45 (83.3) | 42 (55.3) | 13 (15.9) | 100 (47.2) |
| *No* | 9 (16.7) | 34 (44.7) | 69 (84.1) | 112 (52.8) |
| ***Number of antenatal visits*** | | | | |
| *0* | 1 (1.9) | 1 (1.3) | 1 (1.2) | 3 (1.4) |
| *1-3* | 15 (27.8) | 20 (26.3) | 24 (29.3) | 59 (27.8) |
| *4+* | 38 (70.4) | 55 (72.4) | 57 (69.5) | 150 (70.8) |
| ^a^ Complicated birth is defined in Additional file 4 | | | | |
