# Supplementary material for: Facilitating better postnatal care with women-held documents in The Gambia: a mixed-methods study
Source: BMC Pregnancy Childbirth. 2021 Jul 2;21:479. doi: 10.1186/s12884-021-03902-6 (PMC8254330; doi:10.1186/s12884-021-03902-6)
Supplement: Supplementary file 7 — Additional file 7. Content quality and completeness of documents; figures presented as N (%). Results from the assessment of women-held documents. [file 12884_2021_3902_MOESM7_ESM.docx]

| **Additional file 7.** Content quality and completeness of documents; figures presented as N (%) | | | | | |
| --- | --- | --- | --- | --- | --- |
| **Document Content Item** | **Any Document**  **n=211** | **Maternal Record**  **n=207** | **Other discharge-specific documents^a^**  **n=80** | **Prescription card**  **n=111** | **Other documents^b^**  **n=31** |
| 1. Date (of discharge) | 194 (91.9) | 128 (61.8) | 77 (96.3) | 108 (97.3) | 15 (48.4) |
| 2. Mother’s name | 210 (99.5) | 206 (99.5) | 75 (93.8) | 106 (95.5) | 27 (87.1) |
| 3. Date and time of delivery | 198 (93.8) | 193 (93.2) | 11 (13.8) | 2 (1.8) | 15 (48.4) |
| 4. Parity | 205 (97.2) | 205 (99.0) | 1 (1.3) | 1 (0.9) | 2 (6.5) |
| 5. Gravida | 202 (95.7) | 202 (97.6) | 3 (3.8) | 1 (0.9) | 2 (6.5) |
| 6. Relevant obstetric history | 193 (91.5) | 193 (93.2) | 5 (6.3) | 7 (6.3) | 3 (9.7) |
| 7. Mode of delivery | 192 (91.0) | 186 (89.9) | 12 (15.0) | 40 (36.0) | 9 (29.0) |
| 8. Complications / details of birth | 177 (83.9) | 170 (82.1) | 27 (33.8) | 26 (23.4) | 9 (29.0) |
| 9. Birth defects / syndromes | 94 (44.5) | 75 (36.2) | 28 (35.0) | 0 (0.0) | 2 (6.5) |
| 10. Blood loss | 31 (14.7) | 30 (14.5) | 1 (1.3) | 0 (0.0) | 0 (0.0) |
| 11. Apgar score | 157 (74.4) | 153 (73.9) | 21 (26.3) | 1 (0.9) | 1 (3.2) |
| 12. Baby’s gender | 194 (91.9) | 185 (89.4) | 41 (51.2) | 1 (0.9) | 13 (41.9) |
| 13. Birth weight | 196 (92.9) | 193 (93.2) | 40 (50.0) | 1 (0.9) | 12 (38.7) |
| 14. Place of birth | 195 (92.4) | 191 (92.3) | 26 (32.5) | 7 (6.3) | 12 (38.7) |
| 15. Name of staff who delivered | 189 (89.6) | 187 (90.3) | 2 (2.5) | 1 (0.9) | 5 (16.1) |
| 16. Name of staff who issued document | 67 (31.8) | 5 (2.4) | 40 (50.0) | 25 (22.5) | 2 (6.5) |
| 17. Tests / scans / treatment | 162 (76.8) | 158 (76.3) | 8 (10.0) | 3 (2.7) | 7 (22.6) |
| 18. Tests / scans / treatment results | 133 (63.0) | 127 (61.4) | 5 (6.3) | 2 (1.8) | 6 (19.4) |
| 19. HIV status | 4 (1.9) | 4 (1.9) | 0 (0.0) | 0 (0.0) | 0 (0.0) |
| 20. Contraception | 172 (81.5) | 172 (83.1) | 0 (0.0) | 1 (0.9) | 0 (0.0) |
| 21. Vaccination | 17 (8.1) | 6 (2.9) | 0 (0.0) | 0 (0.0) | 12 (38.7) |
| 22. Mother’s age | 210 (99.5) | 205 (99.0) | 66 (82.5) | 109 (98.2) | 16 (51.6) |
| 23. Address | 207 (98.1) | 203 (98.1) | 52 (65.0) | 105 (94.6) | 16 (51.6) |
| 24. Date of next appointment | 76 (36.0) | 45 (21.7) | 32 (40.0) | 2 (1.8) | 3 (9.7) |
| 25. Location of next appointment | 4 (1.9) | 1 (0.5) | 3 (3.8) | 0 (0.0) | 0 (0.0) |
| 26. Medications | 198 (93.8) | 184 (88.9) | 7 (8.8) | 110 (99.1) | 3 (1.4) |
| Document is illegible | 22 (10.4) | 13 (6.3) | 6 (7.5) | 4 (3.6) | 1 (3.2) |
| ^a^ Other discharge-specific documents include the discharge card and the discharge checklist | | | | | |
| ^b^ Other documents include scraps of paper, referral forms, ultrasounds and other miscellaneous papers | | | | | |
|  | | | | | |
