# Supplementary material for: Facilitating better postnatal care with women-held documents in The Gambia: a mixed-methods study
Source: BMC Pregnancy Childbirth. 2021 Jul 2;21:479. doi: 10.1186/s12884-021-03902-6 (PMC8254330; doi:10.1186/s12884-021-03902-6)
Supplement: Supplementary file 8 — Additional file 8. Questionnaire responses. Results from the questionnaire each woman completed. [file 12884_2021_3902_MOESM8_ESM.docx]

Additional file 8: Questionnaire responses

| **Questions** | **Complicated birth ^a^** | | **Total (n=212)** | |
| --- | --- | --- | --- | --- |
|  | **Yes (n=100)** | **No (n=112)** |  |  |
| ***What was the reason for your admission to the hospital?*** | | | | |
| Referred by HCP | 62 (62.0) | 3 (2.7) | 65 (30.7) | |
| Self-admitted | 36 (36.0) | 107 (95.6) | 143 (67.4) | |
| Other | 2 (2.0) | 2 (1.8) | 4 (1.9) | |
| ***What was the first symptom that happened to make you see a HCP? ^b^*** | | | | |
| In labour | 62 (62.0) | 86 (76.8) | 148 (69.8) | |
| Water broke | 45 (45.0) | 53 (47.3) | 98 (46.2) | |
| Pain | 76 (76.0) | 105 (93.8) | 181 (85.4) | |
| Bleeding, vomiting, dizzy or felt unwell | 57 (57.0) | 37 (33.0) | 94 (44.3) | |
| Told, tests or planned | 10 (10.0) | 1 (0.1) | 11 (5.2) | |
| Other ^c^ | 7 (7.0) | 0 (0.0) | 7 (3.3) | |
| ***Where have you visited to get advice before coming to the hospital? ^b^*** | | | | |
| Went straight to hospital | 33 (33.0) | 105 (93.8) | 138 (65.1) | |
| Outpatients | 4 (4.0) | 3 (2.7) | 7 (3.3) | |
| Another hospital | 16 (16.0) | 0 (0.0) | 16 (7.5) | |
| Minor health centre | 46 (46.0) | 2 (1.8) | 48 (22.6) | |
| Other ^d^ | 8 (8.0) | 2 (1.8) | 10 (4.7) | |
| ***Who did you see at that contact? ^b^*** | | | | |
| TBA | 2 (3.0) | 0 (0.0) | 2 (2.7) | |
| Community health nurse | 19 (28.4) | 1 (16.7) | 20 (27.0) | |
| Midwife | 17 (25.4) | 2 (33.3) | 19 (25.7) | |
| Nurse or doctor | 39 (58.2) | 3 (50.0) | 42 (56.8) | |
| Other ^e^ | 2 (3.0) | 0 (0.0) | 2 (2.7) | |
| Not applicable | 33 (33.0) | 105 (93.8) | 138 (65.1) | |
| ***Was it this contact that told you to come to hospital?*** | | | | |
| Yes | 65 (97.0) | 6 (100.0) | 71 (97.3) | |
| No | 2 (3.0) | 0 (0.0) | 2 (2.7) | |
| Missing | 0 (0.0) | 1 (0.9) | 1 (0.5) | |
| Not applicable | 33 (33.0) | 105 (93.8) | 138 (65.1) | |
| ***Who sent you to this hospital?*** | | | | |
| No one; self-referred | 8 (8.0) | 49 (43.8) | 57 (26.9) | |
| The outpatient department or another hospital | 21 (21.0) | 8 (7.1) | 29 (13.7) | |
| Minor health centre | 53 (53.0) | 18 (16.1) | 71 (33.5) | |
| Family / friends | 14 (14.0) | 36 (32.1) | 50 (23.6) | |
| Other ^f^ | 4 (4.0) | 1 (0.9) | 5 (2.4) | |
| ***How many other contacts with health services have you had before this visit? ^g^*** | | | | |
| 0 | 2 (2.0) | 0 (0.0) | 2 (1.0) | |
| 1 to 3 | 25 (25.3) | 34 (30.4) | 59 (28.0) | |
| 4 or more | 72 (72.7) | 78 (69.6) | 150 (71.1) | |
| Missing | 1 (1.0) | 0 (0.0) | 1 (0.9) | |
| ***What contact(s) have you had with health services before this visit? ^b^*** | | | | |
| Antenatal clinic | 98 (99.0) | 112 (100.0) | 210 (99.5) | |
| Scans / tests | 89 (89.9) | 87 (77.7) | 176 (83.4) | |
| Hospital admission | 2 (2.0) | 1 (0.9) | 3 (1.4) | |
| Outpatient appointment | 1 (1.0) | 1 (0.9) | 2 (0.9) | |
| Home visit | 0 (0.0) | 0 (0.0) | 0 (0.0) | |
| Traditional birth attendant | 0 (0.0) | 0 (0.0) | 0 (0.0) | |
| Missing | 1 (1.0) | 0 (0.0) | 1 (0.9) | |
| ***Where did your most recent contact prior to this one take place?*** | | | | |
| Minor health centre | 20 (20.0) | 6 (5.4) | 26 (12.3) | |
| Major health centre | 43 (43.0) | 23 (20.5) | 66 (31.1) | |
| Another hospital | 12 (12.0) | 4 (3.6) | 16 (7.5) | |
| This hospital | 21 (21.0) | 74 (66.1) | 95 (44.8) | |
| No prior contact or other ^h^ | 4 (4.0) | 5 (4.5) | 9 (4.2) | |
| ***Did you receive any paper documents from any previous health service contact(s)?*** | | | | |
| Yes, I have it with me | 84 (84.0) | 95 (84.8) | 179 (84.4) | |
| Yes, I handed it to the HCP | 7 (7.0) | 8 (7.1) | 15 (7.1) | |
| Yes, but I nor staff have it | 6 (6.0) | 9 (8.0) | 15 (7.1) | |
| Yes, but I did not bring it | 1 (1.0) | 0 (0.0) | 1 (0.5) ^i^ | |
| No prior contact or wasn’t given one | 2 (2.0) | 0 (0.0) | 2 (0.9) | |
| ***If you brought a document, has a member of staff looked at it and used it?*** | | | | |
| Yes | 98 (100.0) | 112 (100.0) | 210 (100.0) | |
| No | 0 (0.0) | 0 (0.0) | 0 (0.0) | |
| Don’t know | 0 (0.0) | 0 (0.0) | 0 (0.0) | |
| Not applicable | 2 (2.0) | 0 (0.0) | 2 (0.9) | |
| ***Do you plan to see a healthcare provider for a check-up after this hospital visit?*** | | | | |
| Yes | 99 (99.0) | 112 (100) | 211 (99.5) | |
| No | 1 (1.0) | 0 (0.0) | 1 (0.5) | |
| ***Where do you plan to go for that check-up? ^b^*** | | | | |
| This hospital | 45 (45.5) | 80 (71.4) | 125 (59.2) | |
| Another hospital | 10 (10.1) | 2 (1.8) | 12 (5.7) | |
| Health centre | 38 (38.4) | 23 (20.5) | 61 (28.9) | |
| Antenatal clinic | 11 (11.1) | 7 (6.3) | 18 (8.5) | |
| Other ^j^ | 2 (2.0) | 2 (1.8) | 4 (1.9) | |
| Not applicable | 1 (1.0) | 0 (0.0) | 1 (0.5) | |
| ***How will you tell them what happened here in the hospital? ^b^*** | | | | |
| Discharge documents given from HCP | 80 (80.0) | 62 (55.4) | 142 (67.0) | |
| I wrote it down so I can tell staff I see in the future | 0 (0.0) | 0 (0.0) | 0 (0.0) | |
| HCP told me so I can tell other staff | 17 (17.0) | 15 (13.4) | 32 (15.1) | |
| I asked the HCP to tell me/family/friend | 3 (3.0) | 1 (0.9) | 4 (1.9) | |
| HCP is sending it to the next staff I am seeing | 1 (1.0) | 0 (0.0) | 1 (0.5) | |
| I do not know | 12 (12.0) | 28 (25.0) | 40 (18.9) | |
| Other | 9 (9.0) | 19 (17.0) | 28 (13.2) | |
| ***How much does the woman understand about what happened at the hospital?*** | | | | |
| Understands almost all things | 70 (70.0) | 97 (85.8) | 167 (78.8) | |
| Broadly understands events | 19 (19.0) | 6 (6.2) | 25 (11.8) | |
| Understands only the basic events | 5 (5.0) | 7 (6.2) | 12 (5.7) | |
| Understands very little | 6 (6.0) | 2 (1.8) | 8 (3.8) | |
| ***Do you think it is important to get written information about what has happened to you? ^b^*** | | | | |
| Yes, but I do not know why | 4 (3.4) | 5 (4.5) | 9 (4.2) | |
| Yes, it helps with postnatal appointments | 79 (79.0) | 96 (85.7) | 175 (82.5) | |
| Yes, I get attended to faster | 1 (1.0) | 1 (0.9) | 2 (0.9) | |
| Yes, it feels more professional | 1 (1.0) | 0 (0.0) | 1 (0.5) | |
| Yes, it gives me more confidence about what to do | 15 (15.0) | 9 (8.0) | 24 (11.3) | |
| Yes, for other reason | 4 (4.0) | 4 (3.6) | 8 (3.8) | |
| No, but I do not know why | 1 (1.0) | 1 (0.9) | 2 (0.9) | |
| No, the note gets lost | 0 (0.0) | 0 (0.0) | 0 (0.0) | |
| No, everyone receives same care | 0 (0.0) | 1 (0.9) | 1 (0.5) | |
| No, for other reason | 5 (5.0) | 5 (4.5) | 10 (4.7) | |
| I do not know if it is important | 2 (2.0) | 3 (2.7) | 5 (2.4) | |
| ^a^ Complicated birth is defined in Additional file 4  ^b^ Multiple response question – total may not add to 100%  ^c^ Other includes previous medical history  ^d^ Other includes outreach station, traditional healer and pharmacist/chemist  ^e^ Other includes pharmacist and village health worker  ^f^ Other includes traditional birth attendant and pharmacist/chemist  ^g^ Responses do not include contacts made with traditional healers  ^h^ Other includes home visit and outreach station  ^I^ One woman didn’t bring her documents because she had lost them  ^j^ Other includes outreach station, traditional healer and pharmacist/chemist  ^d^ Other healthcare provider includes health centres, antenatal clinics, outreach/trekking stations, traditional healers and pharmacists/chemists | | | |  |
