# Supplementary material for: Facilitating better postnatal care with women-held documents in The Gambia: a mixed-methods study
Source: BMC Pregnancy Childbirth. 2021 Jul 2;21:479. doi: 10.1186/s12884-021-03902-6 (PMC8254330; doi:10.1186/s12884-021-03902-6)
Supplement: Supplementary file 9 — Additional file 9. Qualitative participant demographics. Demographic data of the healthcare professionals that participated in the focus group discussions and semi-structured interviews. [file 12884_2021_3902_MOESM9_ESM.docx]

Additional file 9: Qualitative participant demographics(12)

| **Characteristic** | **Categories** | **Number of Participants (%)** |
| --- | --- | --- |
| **Age** | 25 and under | 3 (10) |
|  | 26-35 | 17 (56.6) |
|  | 36-45 | 7 (23.3) |
|  | 46-55 | 2 (6.6) |
|  | 56 and over | 1 (3.3) |
| **Gender** | Male | 10 (33.3) |
|  | Female | 20 (66.6) |
| **Profession** | Doctor | 8 (26.6) |
|  | Midwife | 13 (43.3) |
|  | Nurse | 9 (30) |
| **Years of clinical experience** | 0-5 | 15 (50) |
|  | 6-10 | 5 (16.6) |
|  | 11-20 | 8 (26.6) |
|  | 21 and over | 2 (6.6) |
| **Religion** | Muslim | 25 (83.3) |
|  | Christian | 5 (16.6) |
| **Ethnic group / tribe** | Mandinka | 10 (33.3) |
|  | Fula | 8 (26.6) |
|  | Wolof | 3 (10) |
|  | Manjago | 3 (10) |
|  | Aku | 2 (6.6) |
|  | Serahuli | 1 (3.3) |
|  | Serer | 1 (3.3) |
|  | Jola | 1 (3.3) |
|  | Tukulor | 1 (3.3) |
