# Supplementary material for: Facilitating better postnatal care with women-held documents in The Gambia: a mixed-methods study
Source: BMC Pregnancy Childbirth. 2021 Jul 2;21:479. doi: 10.1186/s12884-021-03902-6 (PMC8254330; doi:10.1186/s12884-021-03902-6)
Supplement: Supplementary file 10 — Additional file 10. Barriers and facilitators for effective handover of information from HCPs to other HCPs and women. Results from the focus groups discussions and semi-structured interviews. [file 12884_2021_3902_MOESM10_ESM.docx]

**Additional file 10.** Barriers and facilitators for effective handover of information from HCPs to other HCPs and women

| Theme | Category | Example |
| --- | --- | --- |
| HCPs role in completing and handing over documents to women | **Facilitators:** |  |
|  | HCPs habit of completing the maternal records | *“they will go with their antenatal card… you know their antenatal card continues up til… the end of the puerperium, which is 6 weeks post-delivery…”* [M] [2]  *”every woman should have a card, an antenatal card, the yellow one”* [D] [3] |
|  | HCPs knowledge of what should be recorded on the documents | *“These discharge cards have your details; your name, address, age, your date of admission, date of discharge… Whether cs or whether normal delivery, whether anaemia transfused… and we give you appointment date”* [M] [2]  *“We record everything on their antenatal cards, like the weight of the baby, the sex, the APGAR score, the condition of the mother”* [M] [1] |
|  | **Barriers:** |  |
|  | Shortage of HCPs to complete documents | *“most of our wards are understaffed so already they have a lot to fill… They have a checklist that after delivery they need to fill and then they have the book to fill”* [D] [3]  *“Try to increase the number of staff we have on the ground. You don’t expect me to be alone here as a midwife to run both wards. At the end of the day, you expect me to do things effectively”* [M] [3] |
|  | Too many discharge documents for HCPs to complete | *“when they are going, we give them their antenatal cards, their duplicate of the checklist er and er the discharge cards, the green one and the white one”* [M] [3]  *“we give them back their antenatal cards. We give them two discharge cards, one for follow-up and the other one… this, a bit different. This is the one for follow-up.”* [N] [3] |
|  | No protocol for HCPs to follow | *“a standardised protocol with written documents and everything, from the Ministry, it has to be a national er po-policy, you understand, and that policy needs to be implemented.”* [D] [1] |
| Ability of women to utilise the documents | **Barriers:** |  |
|  | Women’s ability to read the information on documents | *“there is a lot of women who didn’t go to school and are illiterate… Right now, the illiterate will be like 60% and 40% will be literate yes.”* [M] [2]  “*Sometimes it is just related to their educational level, whether they are educated or illiterate so*” [D] [1]  *“Yes some of them don’t understand and then some of them are-because of er er um how do you call it… illiteracy or something I think?”* [D] [1] |
|  | Women losing or forgetting the documents | *“I think er it is a societal culture because um people have problems of keeping documents. They fold it and fold it and fold it to a way that at the end of the day”* [D] [1]  *“Sometimes they forget to bring-when especially when it’s not stapled together, they forget to bring their antenatal card, they will just bring their discharge cards.”* [M] [3]  “*But sometimes, they have just an urgent thing or an urgent- er a new emerging problem and if they rush to the hospital, they forget everything*.” [D] [3] |
| The code letter after each quotation refers to the cadre of HCP; doctor (D), midwife (M) and nurse (N) and the code number represents the hospital in which they worked (1, 2 or 3 in relation to the supplementary materials). | | |
